# Concurrence of Adenomyoepithelioma of the Breast and Gastrointestinal Stromal Tumor of the Stomach: A Case Report and Review of the Literature

**DOI:** 10.3390/medsci11030057

**Published:** 2023-09-10

**Authors:** Fatma Althoubaity, Lamar A. Wazira, Hanin M. Y. Ahmad, Reyof T. Aljuhani

**Affiliations:** 1 Department of Surgery, King Abdulaziz University Hospital, Jeddah 21589, Saudi Arabia; 2 Department of Surgery, Collage of medicine, King Abdulaziz University, Jeddah 21589, Saudi Arabia

**Keywords:** Adenomyoepithelioma (AME), ductal carcinoma in situ (DCIS), gastrointestinal stromal tumors (GISTs), coexistence, immunohistochemistry, histology

## Abstract

Adenomyoepithelioma (AME) of the breast and gastrointestinal stromal tumors (GISTs) are rare benign (primarily) tumors observed in the breast and gastrointestinal tract, respectively. The coexistence of both of these rare tumors is extremely rare; therefore, the author describes the clinical presentation and pathophysiological findings of such a unique case in this study. A 56-year-old female patient with no medical history presented with a substantial right breast lump, severe nausea, and vomiting, and suffered from iron deficiency anemia. Radiological observation and a right breast excisional biopsy diagnosed the patient with AME associated with ductal carcinoma in situ (DCIS). Endoscopy and a CT scan of the stomach revealed the existence of GIST. This is the first reported case of concurrence of a huge mass of AME and GIST in a patient. Histological and immunohistochemistry tests using p63, SMA, calponin, and Ki67 markers for the breast tumor and DOG-1, CD34, and CD117 markers for the gastric tumor revealed the non-invasive benign state. The patient had a right breast mastectomy with a negative resection margin. AME of the breast and GIST pose diagnostic challenges due to their erratic morphological characteristics and can cause misinterpretation drawn solely from radiological tests. Effective and accurate diagnostics require assessing the histological and immunohistochemistry findings of the tumor to identify the invasiveness of the neoplasm and the associated risk levels. This report, thus, creates awareness among clinicians and pathologists for the consideration of such possibilities and, therefore, conducts the necessary diagnostics and prophylactic treatments.

## 1. Introduction

Adenomyoepithelioma (AME) is a rare, uncommon benign tumor of the breast and was first characterized by Hamperl in 1970. In 2019, the World Health Organization defined AME as a biphasic neoplasm with a characteristic enlarged and detectable proliferation of luminal and myoepithelial cells [1,2,3]. AME is difficult to diagnose through imaging techniques and may produce misinterpretation. Therefore, the intervention of histological and immunohistochemistry tests is required for the final diagnosis [4]. Ductal carcinoma in situ (DCIS) is the malignant proliferation of cells of the basement membrane in the mammary duct. Past research has reported a few cases of the coexistence of AME with DCIS, and both conditions require surgical intervention with proper excision of the tumor [4,5,6,7,8,9,10,11,12].

Even though gastrointestinal stromal tumors (GISTs) have a <1% prevalence rate, they are the most common mesenchymal neoplasm in the gastrointestinal tract originating from Cajal cells of the myenteric plexus [13]. GIST is also primarily benign, but 10–30% of cases in the literature have reported malignancies. Histologically, GISTs can be of a spindle cell type (70%), epithelioid type (20%), and mixed type (10%) [14]. GISTs are diagnosed using imaging or endoscopy and confirmed using immunohistochemistry and morphological studies [15,16]. The coincidence of GIST with AME is rare, and only one case has been reported so far [17].

Here, we report a unique case of a 56-year-old woman who presented with a concurrent large mass in the right breast diagnosed with AME post-mastectomy and a posterior gastric tumor mass of spindle cell-type GIST.

## 2. Case Presentation

**Figure 1:**
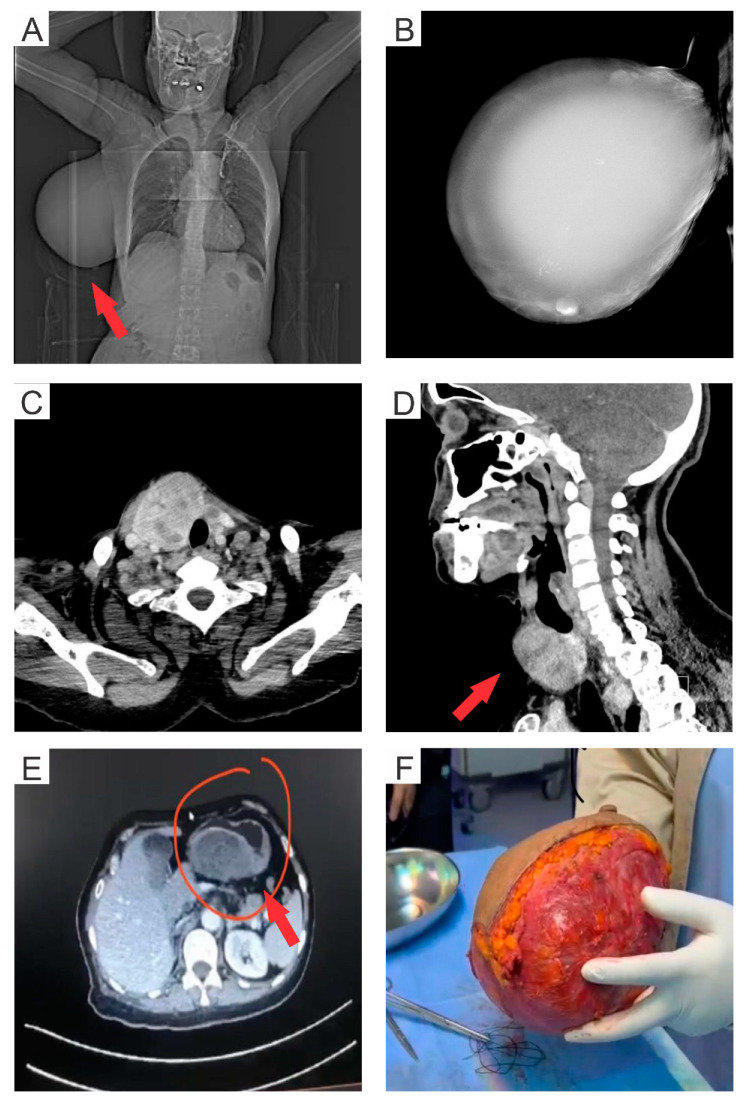
(**A**) Computed Tomography (CT) image of the chest showing a large mass (indicated by the red arrow) in the right breast, (**B**) Mammographic image of the right breast displaying a large dense mass occupying the entire breast, (**C**) CT scan showing right breast mass with multiple areas of intrinsic coarse as well as finer calcifications especially superiorly and inferiorly. Also, it abuts the right pectoral muscle with a preserved fat plane in between. (**D**) CT image of the neck demonstrating a large right thyroid lobe (indicated with the red arrow) with multiple heterogenous thyroid nodules, (**E**) CT image of the abdomen showing a large gastric mass of 17 cm (indicated by red arrow), and (**F**) Surgically excised tumor mass from the right breast.

A 56-year-old multiparous Saudi female, having no family or personal history of breast cancer, was diagnosed with a huge, non-tender, painless mass covering the entire right breast. The mass was observed 15 years ago, but the patient did not consult a doctor for treatment. Later on, the patient also suffered from profound iron deficiency anemia. She also had a large posterior gastric wall mass identified four years ago in abdominal-computed tomography (CT) but refused endoscopy. Recently, the patient reportedly suffered from severe nausea and vomiting. The CT of the abdomen displayed a large gastric mass of 17 cm. An upper gastrointestinal tract (GIT) endoscopy, and a cold forceps biopsy, were advised to evaluate the severity and status of the detected mass (Figure 1E). The endoscopy revealed a large, fungating, ulcerated, non-circumferential mass (involving one-half of the lumen circumference). The tumor mass extended from the lesser curvature of the stomach to the duodenal bulb. Pathological examination of the biopsy sample of the gastric mass confirmed the presence of GIST with a spindle cell-type histology. The patient was advised on a daily medication of 400 mg of Imatinib Mesylate for six weeks as a neoadjuvant approach to reduce the tumor size. Post-treatment, the gastric tumor was surgically excised by total gastrectomy with esophagojejunostomy.

A chest CT was performed as the breast tumor mass was huge, and to rule out any chest wall involvement, since the initial clinical impression was of cytosarcoma or phyllodes tumor (Figure 1A), and radiologists prefer to take patients for exscional biopsies. Further, a mammogram of the right breast showed a 16 × 11 cm well-circumscribed large dense mass replacing the entire breast with inferior displacement of the right nipple (Figure 1B). The mass demonstrated coarse and fine calcifications at multiple areas, especially superiorly and inferiorly. The mass abutted the right pectoral muscle in between a preserved fat plane, as observed in the CT scan (Figure 1C). The breast tumor was operated on with a simple right mastectomy and right sentinel lymph node biopsy. The excised mass measured a length of 19 cm from medial to lateral, 13 cm from superior to inferior, and 10 cm from anterior to posterior, covered with an unremarkable ellipse of the skin (19 × 11.5 cm), including a nipple–areolar complex of 3 × 5 cm dimension (Figure 1F). The tumor was serially sectioned from lateral to medial into eight slices, revealing a large cystic lesion involving the entire breast, filled with clear yellow fluid. The cystic lining was smooth and showed the presence of two solid polyploidal lesions within the cyst. The cyst wall thickness ranged from 0.4–0.7 cm. Further, a CT scan of the neck demonstrated a large right thyroid lobe with multiple heterogenous thyroid nodules, which will need further evaluation through fine needle aspiration (Figure 1D). Later on, the thyroid mass will be operated using thyroidectomy.

### Histology and Immunohistochemistry

Tissue samples extracted from resected breast tumors and GIST mass were processed for histological examination. A number of 4 μm thick paraffin sections were dissected from these tissue samples, later fixed in formalin, and washed. These paraffin sections were stained with hematoxylin and eosin solution and analyzed under a digital light microscope (Olympus BX50, Japan). Histological sections from the right breast mass showed a large cystic wall lined by a benign biphasic proliferation of epithelial and myoepithelial cells with focally lining and containing lobulated, polypoidal, well-circumscribed neoplastic growth. Scattered foci of atypical epithelial cell proliferation were identified with no infiltrative or destructive growth. The immunohistochemistry of these formalin-fixed paraffin tissue sections from the breast tumor and GIST mass was performed in Ventana Benchmark XT (Ventana Medical Systems, Inc., USA) using the avidin–biotin complex method. The formalin-fixed paraffin tissue samples showed positive p63 and actin (SMA) markers highlighting the proliferating myoepithelial cells, and were negative in the proliferating atypical epithelial cells. Calponin-positive staining highlighted the myoepithelial cells. HER2-stained cells showed incomplete weak membranous staining in 60% of tumor cells and complete, weak membranous staining in 10% of tumor cells. DCIS was found in 9 out of 23 blocks examined (~5%) as scattered foci sparing two areas (0.3 cm and 2.8 cm) with low volume in a contiguous pattern. However, the predominant patterns were solid and cribriform. DCIS and benign breast tissue also displayed epithelial ductal hyperplasia and microcalcifications. Lobular involvement, necrosis, inflammatory lymph nodes, and fibrocystic changes were not observed. Nipples, skin, skeletal muscles, and lymph nodes were all negative for malignancy. DCIS was positive for estrogen receptors with strong nuclear positivity in 90% of tumor cells. On the other hand, it was negative for both progesterone receptor and HER2, and Ki67 was ~1%. The histological and biomarker pattern observed in the breast mass indicated non-invasive benign cystic adenomyoepithelioma involving scattered small foci of DCIS, nuclear grade 2 within the epithelial component. Further, DCIS was not observed in the surrounding breast tissue. Radiological measurements of the 16 cm gastric tumor identified the risk levels to be moderate to high. The histological section of the tumor displayed no mitotic figures. The immunohistochemistry analysis of the gastric mass was positive for DOG-1, CD34, and CD117 markers and Desmin-negative cells. Germline genetic testing of the GIST samples for mutation identification was not performed. Overall, the pathological results supported the presence of a benign gastrointestinal stromal tumor with a spindle cell-type morphology.

The patient’s condition was stable after mastectomy and had a smooth post-operative course with no complications. Still, our patient needs management for her gastric and thyroid masses, and she is on follow-up in our surgical clinic. The patient is recommended for a post-operative follow-up of one month, followed by a quarterly visit in the first year, half-yearly in the second year, and then annually later. The patient’s status will be monitored through breast imaging.

## 3. Discussion

AME is an uncommon neoplasm of the breast and is primarily benign; however, several cases have also reported malignancies. It poses clinical and diagnostic challenges due to the morphological heterogeneity in clinical presentation observed in the imaging techniques and, thus, cannot be solely used for accurate interpretations. AME occurrence shows female predominance, with most patients being elderly or middle-aged (mean age 59 years). However, diversity in the age spectrum of patients has been reported, with the youngest being an adolescent girl of 14 years and as elderly as 82 years [18,19]. Variations in the age of patients presenting AME could result from the divergence of screening and diagnostic modalities worldwide. In Table 1, we have presented similar cases of AME coexisting with DCIS, as observed in our study.

The present study reports a case of benign AME in a Saudi female aged 56 years, demonstrating a 16 × 11 cm well-circumscribed large painless, tender mass extending over the entire right breast with calcification at multiple areas. The AME tumor in this study measured a dimension of 13 cm × 19 cm × 10 cm, which was significantly higher compared to the two earlier reports from China and India, where the measurements of the tumor were 3.5 cm × 3 cm × 2.2 cm, and 2.5 cm × 2.4 cm × 2.3 cm, respectively [21,22]. Another report by González et al. observed an adenomyoepithelioma tumor of 8–10 cm dimension in a 13-year-old teenage female [19]. In earlier reports, AME of the breast has been observed to coexist with breast cancer [6] (also see Table 1). In this study, we report a similar coexistence of AME with small scattered foci of DCIS, nuclear grade 2 exhibiting epithelial ductal hyperplasia and microcalcifications. The immunohistochemistry assessment of AME is based on the biomarkers CK, AE1/AE3, CK7, CK5/6, and EMA for epithelial components, and calponin, SMA, SMMH, p63, 34E12, and S-100 identifies the myoepithelial components [21,23]. Some studies have also utilized progesterone- and estrogen-receptor positivity as diagnostic markers [18]. This study reports the positivity of the samples for SMA, p63, and calponin biomarkers indicative of the benign AME tumor with lobulated, polypoidal, and well-circumscribed neoplastic growth. It has not transformed into a malignant state. At the same time, in an Australian report, lesions were composed of round glands and solid clusters consisting of both epithelial and myoepithelial cells [9]. In contrast, AlQurashi et al. and Laforga et al. observed transformation into the apocrine metaplasia of the epithelial cells [12,24]. HER2 expression level is a well-established prognostic and predictive marker in invasive breast cancer. Several investigative studies have established a strong correlation between the overexpression of HER2 in DCIS and the clinicopathological molecular markers and parameters associated with aggressiveness and worse prognosis. Furthermore, HER2-positive DCIS has higher incidences of recurrence of in situ and invasive breast cancer in patients [25]. Therefore, HER2-targetted therapy could prove to be clinically relevant in HER2-positive DCIS patients, thus reducing invasiveness and recurrences rates. However, in this study, the in situ DCIS identified in the breast tissues were negative for HER2 biomarkers and Ki67 index was also ~1%.

GIST is the most common mesenchymal neoplasm of the GIT, with the size varying from as small as 1 cm and extending up to 40 cm. Due to its low prevalence (<1%), its etiology and the associated risk factor have not been explored in detail [13,14]. The concurrence of AME and GIST together in a patient is an extremely rare event, and only one such case has been reported by Hegyi et al. in a 41-year-old female patient from the UK [17] (see Table 1). This study reports a similar unique concurrence of benign non-invasive AME and benign GIST in a 57-year-old female. Herein, the gastric mass showed no mitotic figures; however, the tumor on the glass slide section represents far less than the requested 5 mm^2^ surface area for counting mitosis. Further, upon radiological assessment, the gastric tumor mass measured 16 cm in size, allowing us to interpret the prognosis as moderate to high risk. In comparison, a study from the UK reported a solid and peripherally enhancing mass of 5 cm × 4 cm in size in the CT scan and a mitotic index of less than 1/50 HPF. Furthermore, the immunohistochemistry pattern demonstrated positivity for CK7, AE1/AE3, CK903, MNF116, CK14, CK5/6, CD117, EMA, SMA, S100 protein, CD10, CD34 and calponin biomarkers, but negative for desmin, CK20 and H-caldesmon [17]. In parallel, our immunohistochemistry data for gastric mass in the present study exhibited positivity for DOG-1, CD34, and CD117 biomarkers and negative for desmin. Also, the Ki67 value was ~1%, indicative of the benign state of the GIST.

Due to the rare occurrence of AME, the treatment protocol is not well established. Adjuvant chemotherapy remains ineffective in the majority of cases. However, cyclophosphamide and eribulin have beneficial roles in malignant AME [26,27]. Thus, surgical excision with the establishment of a negative resection margin remains the mainstream management practice for malignant and benign AME. The treatment protocol employed in the present study involved resection of the AME tumor of the breast using a simple mastectomy. Surgical resection is the gold standard treatment for GIST and has been used in 80% of the cases [14]. Adjuvant and neoadjuvant chemotherapy are other treatment procedures considered in cases where GIST is borderline resectable, metastatic, recurring, or unresectable, and also to reduce morbidity. Chemotherapy uses tyrosine kinase inhibitors such as imatinib, avapritinib, and sunitinib to treat GISTs. Other drugs, such as ponatinib, regorafenib, nivolumab, and ipilimumab, have also demonstrated effectiveness against GIST [28,29]. Gene sequencing of the biopsy sample from the patients can aid in devising effective medication based on the germline mutations. In our study, adjuvant chemotherapy was used against the identified GIST through the administration of imatinib for six weeks, followed by gastrectomy. However, germline genetic testing of the GIST tumor samples was not performed. After the surgical excision of the tumor, the resected margins were reported negative for neoplastic lesions. Only one piece of research had a similar coincidence, but the pathogenesis in our case was unknown due to the lack of any chronic illness that can contribute to the development of such tumors.

## 4. Conclusions

This study reports the rarest concurrence of AME of the breast with DCIS and GIST in a patient. This is the second report about the occurrence of such a unique condition. Reporting such cases generates awareness among clinicians and pathologists to assess and devise effective treatment strategies. The invasiveness and risk levels of the tumor are challenging and cannot be interpreted solely the radiological studies. Therefore, it is necessary to perform a biopsy and histopathological tests to accurately assess the prognostic stage of the infection and implement essential treatment strategies. Surgical excision is the best available treatment option to avoid the transformation to malignancies. However, evaluating the surgical margins is the most important to prevent the chances of any residual lesions post-resection.

## Figures and Tables

**Table 1:** Similar cases of coexistence of AME with DCIS and GIST.

Author	Year	Age	Ethnicity	Tumor Characteristics	Management
Naoto Kuroda et al. [7]	2008	66	Japanese	Coexistence of benign AME with DCIS	Partial mastectomy
Jeong S. Han and Yan Peng [8]	2010	55	ND	Coexistence of benign AME with DCIS	Total mastectomy
Sanjay Warrier et al. [9]	2013	55	Australian	Coexistence of benign AME with DCIS	Adjuvant radiotherapy and mastectomy with reconstruction
Hiroyuki Maeda et al. [20]	2013	35	Japanese	Coexistence of benign AME with DCIS	Adjuvant endocrine therapy and mastectomy
Mirei Kamei et al. [6]	2015	71	Japanese	Coexistence of benign AME with DCIS	Mastectomy
Momoko Tokura et al. [10]	2018	68	Japanese	Coexistence of benign AME with DCIS	Partial mastectomy
Gaurav P. Gahlot et al. [11]	2021	51	Indian	Coexistence of benign AME with DCIS	Modified radical mastectomy
Mariam AlQurashi et al. [12]	2022	49	Saudi	Coexistence of benign AME with DCIS	Mastectomy
Hegyi L. et al. [17]	2015	41	Caucasian	Malignant myoepithelioma of the breast and GIST of the small bowel developed in patient with neurofibromatosis type 1 (NF-1)	Right simple mastectomy with excision of the pectoral fascia and GIST excised by limited resection
Present study	2023	56	Saudi	Coexistence of benign AME with DCIS and spindle cell-type GIST	Adjuvant therapy with total gastrectomy and mastectomy

AME: Adenomyoepithelioma; DCIS: Ductal carcinoma in situ; GISTs: Gastrointestinal stromal tumor; ND: Not disclosed.

## Data Availability

Data of patients available in medical records at King Abdulaziz University Hospital with her hospital number and can be reached through author Prof Fatma Al thoubaity.
